# Botanical Origin, Pollen Profile, and Physicochemical Properties of Algerian Honey from Different Bioclimatic Areas

**DOI:** 10.3390/foods9070938

**Published:** 2020-07-16

**Authors:** Mounia Homrani, Olga Escuredo, María Shantal Rodríguez-Flores, Dalache Fatiha, Bouzouina Mohammed, Abdelkader Homrani, M. Carmen Seijo

**Affiliations:** 1 Laboratory of Sciences and Technics of Animal Production (LSTPA), Abdelhamid Ibn Badis University (UMAB), 27000 Mostaganem, Algeria; mounia-homrani@hotmail.fr (M.H.); fdalache2@yahoo.fr (D.F.); abdelkader.homrani@univ-mosta.dz (A.H.); 2 Department of Vegetal Biology and Soil Sciences, Faculty of Sciences, University of Vigo, As Lagoas, 32004 Ourense, Spain; oescuredo@uvigo.es (O.E.); mariasharodriguez@uvigo.es (M.S.R.-F.); 3 Laboratory of Vegatal Protection, Abdelhamid Ibn Badis University (UMAB), 27000 Mostaganem, Algeria; lvp@univ-mosta.dz

**Keywords:** characterization, Algerian honey, botanical origin, biogeographical origin

## Abstract

The palynological and physicochemical analysis of 62 honey samples produced in different biogeographical areas of Algeria was conducted. Results showed high variety in the botanical origin of samples and their physicochemical profile. Twenty-six samples were polyfloral honey, 30 were unifloral honey from different botanical sources such as *Eucalyptus*, *Citrus*, *Apiaceae*, *Punica*, *Erica*, *Rosmarinus*, *Eriobotrya*, or *Hedysarum*, and 6 were characterized as honeydew honey. Pollen analysis allowed the identification of 104 pollen types belonging to 51 botanical families, whereas the physicochemical profile showed important variations between samples. Multivariate techniques were used to compare the characteristics of samples from different biogeographical areas, showing significant differences between humid-area samples, located in the northeast of the country, and samples taken in semiarid, subhumid, and arid zones. Principal-component analysis (PCA) extracted nine components explaining 72% of data variance, being 30%, the sum of Component 1 and Component 2. The plot of both components showed samples grouped upon botanical and geographical origin. The results of this paper highlighted the great variability in honey production of Algeria, evidencing the importance of honey characterization to guarantee authenticity and to valorize local production.

## 1. Introduction

Beekeeping in Algeria is part of agricultural life. It is practiced in mountainous regions such as the Aures Mountains, Kabylie, and Dahra, in the coastal plains, and the valleys of the big wadis, but it is more intensive in the northern part of the country where the flora provides resources for honey throughout most of the year [1]. Beekeeping plays an important role in the rural areas of the Mediterranean region due to the suitable climatic conditions and high biodiversity. Highly valued honey supplies are a welcome income for farmers and hobby beekeepers [2].

The composition of this food, obtained by bees from nectar, honeydew, or both sugary resources, is highly dependent on honeybee activity and the biogeography of the area in which it was produced. So, physicochemical properties such as color, pH, electrical conductivity, sugar content, organic acids, ash content, polyphenols, flavonoids, and other phytochemicals differ with different environmental conditions and plant communities visited by honeybees [3].

The Mediterranean region of Algeria is an outstanding biogeographical crossroads that resulted from a complex history and highly heterogeneous environmental factors [4]. Algeria has significant plant biodiversity, with about 3150 species [5], of which more than 500 species are endemic and characteristic of the Mediterranean basin, the Atlas Mountains, the high steppe plateau, the large Saharan plateau, and the mountainous massifs of the Algerian Sahara [6]. The melliferous area in Algeria is estimated at 797,122 hectares with a predominance of forests and maquis, natural meadows, orchards with orange fruits, and other crops.

The main honey sources in the area are trees like *Eucalyptus* and some wild herbaceous plants, mainly from the *Apiaceae* (*Foeniculum*, *Daucus*, *Coriandrum*, *Eryngium*, *Pimpinella*), Asteraceae (*Galactites tomentosa*, *Centaurea*, *Echinops*), *Brassicaceae* (*Brassica* and *Sinapis*), and *Fabaceae* (*Hedysarum*, *Ononis natrix*, *Trifolium*, *Melilotus*) families [7,8,9]. Some plants, such as *Ziziphus lotus*, or grown plants, such as *Helianthus annuus* or *Citrus*, were also named as important plants sources for honey production [10]. In this context, the main honey types produced in the country are *Eucalyptus*, *Citrus*, forest (honeydew), jujube, sunflower, rosemary, and wild mustard. However, the production of other rare or less known honey types was mentioned in some papers [11,12].

The country is considered a traditional consumer of honey, but national production does not achieve self-sufficiency, so to cover the relevant needs, large quantities of honey are imported every year from countries such as China, India, and Saudi Arabia. The lack of national legislation and unknowns about Algerian honey characteristics have a negative impact on the scarce development of Algerian beekeeping. In the absence of specific legislation, there are also no requirements to assess the quality or geographical and botanical origin of honey in the market. Therefore, consumers and local producers are not protected against frauds, and honey of good quality is not valorized. Improving knowledge in the characteristics of local production is the main course of action for their valorization and to link agricultural products with the territory in which they were produced. These studies facilitate the recognition of specific properties with botanical origin and allow differentiating local production from imported honey. Currently, there is no designation of origin for local honey that would contribute to valorizing Algeria honey production.

Some authors studied the characteristics of honey produced in Algeria and found differing results as to the pollen content [8,9,10,13], physicochemical attributes and pollen profile of samples from different areas [7,14,15,16], their botanical origin [9,11,17,18,19], antibacterial and antioxidant activity [20], or specific compounds such as sugars and phenolic or volatile compounds [21,22]. However, honey production in Algeria remains poorly studied. On the basis of these facts, this paper aimed to provide scientific information on the botanical origin and physicochemical profile of honey samples from different bioclimatic areas of Algeria.

## 2. Material and Methods

### 2.1. Study-Area Bioclimatology

The territory of Algeria is composed of four principal structural units. Near the coast, in the north, there is a narrow region with several hills and plains named Tell. This region is densely populated and constitutes the main agricultural land in the country. It has a Mediterranean climate with mild winters and moderate rainfall. Mean annual temperatures are close to 22 °C in summer and 10 °C in winter. Rainfall is abundant along this coastal area, increasing the amount of annual precipitation from 400 to 670 mm in the west to near 1000 mm in the east. These weather conditions determined a subhumid-humid climate tendency.

In the central area, several mountain chains were determined as the high plateau area that separates the Mediterranean area from the Saharan Atlas and the desert. This is a highly diverse area dominated in the plains by steppelike morphology. This region is characterized by a semiarid-to-arid climate with irregular and low precipitation (200–400 mm); its bowl profile explains the presence of many salt lakes (called “chotts”) collecting surface water. It continues to the south with the Saharan Atlas that delimits the Sahara desert. The Saharan Atlas is formed by three massifs that are the source of watercourses that supply the wells of oases along the northern edge of the desert; the massifs are Biskra, Laghouat, and Béchar. The Sahara is a windy and very arid area with a continental climate, high thermal amplitude, and extremely poor precipitation of no more than 130 mm.

### 2.2. Honey Samples

**Figure 1:**
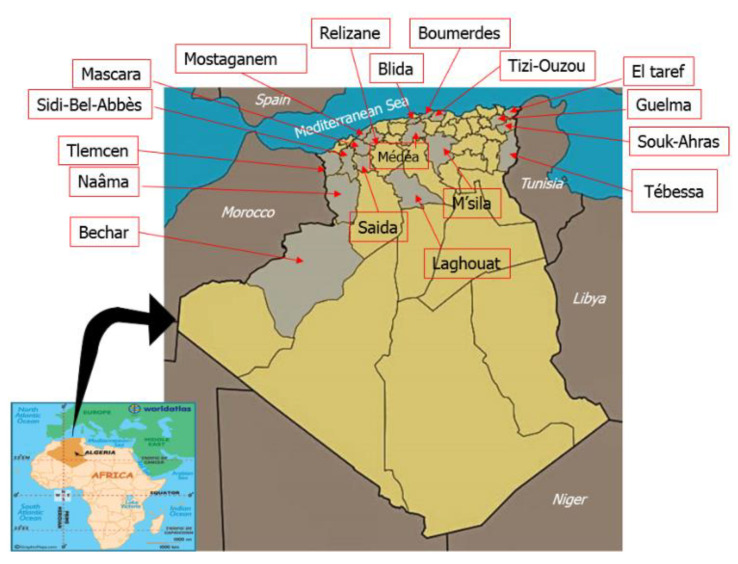
Geographical areas where the samples were taken.

We collected 62 honey samples from different bioclimatic areas of Algeria (Table 1). Concretely, 34 samples were from the semiarid area, 15 were obtained in subhumid areas, 4 in humid areas, and 9 samples in the arid areas of the Sahara and Saharan Atlas (Figure 1). The samples were obtained directly from beekeepers during the 2015–2016 period and were stored refrigerated at 4 °C until analysis. All determinations were carried out after sample homogenization.

### 2.3. Melissopalynological Analysis

#### 2.3.1. Quantitative Pollen Analysis

Ten grams of honey were weighed and dissolved in 20 mL of distilled water. The solution was centrifuged for 10 min at 4500 rpm, and the supernatant was removed until a final volume of 5 mL. The sediment was stirred, and then two aliquots of 10 µL were deposited over a slide to microscopically examine (Nikon UK Ltd., Surbiton, UK). All pollen in the aliquot was counted. The results are expressed as the number of pollen grains per gram of honey considering the average value of the two aliquots.

#### 2.3.2. Qualitative Pollen Analysis

Ten grams of honey were weighed and fully dissolved in 20 mL of distilled water. The solution was centrifuged for 10 min at 4500 rpm, and the supernatant was drawn off. The sediment was again washed with distilled water, and another centrifugation in the same conditions was done. Then, two aliquots (100 µL) of the sediment were deposited into a slide over a heated surface until desiccation. Lastly, drops were covered with a 24 × 24 mm cover glass containing a drop of glicerogelatin with fuchsine.

Slide observation was carried out with a light microscope at 400× or 1000×, as appropriate to improve the identification of pollen types. For each honey sample, at least 500 pollen grains were counted. All pollen grains were classified as the level of pollen type due to difficulties in accessing information about the wild botanical species of apicultural interest in a large part of the territory.

The relative frequency, expressed as a percentage, of all identified pollen type was considered for the pollen spectra of the samples. The following frequency classes were used to establish the representation of pollen types: dominant pollen (≥45%), accompanying pollen (from ≥15% to <45%), important pollen (from ≥3% to <15%), minor pollen (from ≥1% to <3%) and present pollen (≤1%). However, for unifloral honeys, the percentage of the main pollen type was calculated excluding plants considered to be non-nectariferous species, namely, *Acacia*, *Buxus sempervirens*, *Casuarina*, *Cannabis*, *Chamaerops*, *Cistus*, *Cyperus*, *Olea europaea*, *Papaver rhoeas*, *Paronychia argentea*, *Pinus*, *Pistacia*, *Poaceae*, and *Quercus*.

#### 2.3.3. Physicochemical Analysis

The quality parameters of moisture, electrical conductivity, pH, and hydroxymethylfurfural content were determined following the methodology of the International Honey Commission [23]. All determinations were made in duplicate. The results are expressed as the mean of the obtained values.

Moisture was determined with a Carl Zeiss Jena refractometer (Zeiss, Oberkochen, Germany) by measuring the refractive index at 20 °C. Moisture content was calculated using the Wedmore table, and results were expressed as percentages.

Electrical conductivity was measured at 20 °C in a 20% (*w*/*v*) honey solution (dry-matter basis) in CO_2_-free deionized distilled water with an EUTECH instrument conductivity meter (Thermo Fisher Scientific, Massachusetts, USA), and results were expressed as mS/cm; pH was measured by a pH meter (WTW in Lab pH 750) in a solution containing 10 g of honey in 75 mL of distilled water.

Hydroxymethyfurfural (HMF) content was determined using the White spectrophotometric method. Briefly, 5 g of honey was dissolved in 25 mL of distilled water and transferred to a volumetric flask of 50 mL; then, 0.5 mL of Carrez Solution I and 0.5 mL of Carrez Solution II were added. The final volume of 50 mL was set with distilled water. The honey solution was filtered, and the first 10 mL of the filtrate was rejected. Lastly, aliquots of 5 mL were pipetted into 2 tubes (reference and sample solution). Then, 5 mL of sodium bisulfite solution 0.2% was added to the reference, and 5 mL of water was added to the sample solution. The absorbance of the reference against the sample solution was determined at 284 and 336 nm with a UV-vis spectrophotometer (Fisher Scientific, Leicestershire, UK).

Diastase activity was determined through the amount of starch converted by a honey solution. The absorbance of yielded blue during the reactions was spectrophotometrically determined at 660 nm with a UV-vis spectrophotometer (Jenway 6305 UV-Visible Spectrophotometer, Staffordshire, UK) at different times until an endpoint of less than 0.235. Diastase activity was calculated as diastase index (DI) or grams of starch hydrolyzed at 40 °C each hour per 100 g of honey.

### 2.4. Color Determination

Prior to color determination, all samples were decrystallized and left for 20 min in an ultrasound bath to avoid bubbles. Sample color was determined using a HANNA Honey colorimeter (HANNA Instruments, Bedfordshire, UK). This is an instrument that gives the transmittance of honey using glycerol as a reference. Samples were introduced in square optical cuvettes of 10 mm sides, and the color value was taken directly; results are expressed in mm Pfund.

### 2.5. Determination of Total Polyphenol and Flavonoid Content

The Folin-Ciocalteu spectrophotometric method adapted to honey was used to the quantification of polyphenol content [24]. A UV-vis spectrophotometer (Jenway 6305, Staffordshire, UK) was used for this purpose. Absorbance at 765 nm of a honey solution (0.1 g/mL) that reacted with the Folin-Ciocalteu reactive was determined. Ethanolic solutions of gallic acid in different concentrations (0.01–0.50 mg/mL) were used as a standard to construct the calibration curve. The linearity of the curve was 0.997 (*R*^2^). The polyphenol content of the samples was expressed as gallic acid equivalents in mg/100 g.

Total flavonoid content was measured using a similar spectrophotometric method based on an adaptation of the Dowd method [25]. A solution of aluminum chloride reacted with the flavonoids of the samples, prepared in a concentration of 0.33 g/mL with methanol. Absorbance of yellow yielded by the reaction was measured at 425 nm. Different concentrations of the quercetin flavonoid (0.002–0.01 mg/mL) were employed to construct the calibration curve; linearity was 0.998 (*R*^2^). The flavonoid content of honey samples was expressed as mg equivalent of quercetin per 100 g.

### 2.6. Radical-Scavenging Activity

The method used to evaluate the antioxidant activity of honey is based on the discoloration of a 2,2-diphenyl-1-picrilhidrazil (DPPH) solution. The methodology measures the radical-scavenging activity of a honey solution against DPPH by spectrophotometry [26]. A solution (0.1 g/mL) of each honey sample in methanol was prepared. Then, 0.3 mL of the honey solution was mixed with 2.7 mL of the DPPH solution (6 × 10^−5^ M); a blank was also prepared. The honey sample solution and the blank were maintained in the dark at room temperature for 30 min. Then, absorbance at 517 nm was measured with a UV-vis spectrophotometer (Jenway 6305, Staffordshire, UK). Radical Scavenging Activity (RSA) percentage was calculated considering DPPH discoloration for each sample, tested as follows: RSA = [(AbsB − AbsS)/AbsB] × 100, where AbsB is the absorbance of the blank, and AbsS is the absorbance of the honey sample solution.

### 2.7. Sugar-Composition Analysis

Sugar composition was determined with an ion Dionex ICS-3000 chromatography system (Sunnyvale, CA, USA). The system separated sugars by using an analytical polyvinylidene/polyvinyl benzene CarboPac PA1 column (Dionex 3 × 250 mm) suitable for mono-, di-, and trisaccharides, and oligosaccharide analysis in general, and a pulsed amperometry detector with a gradient of two mobile phases (A and B). Phase A involved ultrapure water, while Phase B involved 200 mM NaOH (HPLC grade, Merck, Kenilworth, NJ, USA). The sugars of honey solutions (10 mg/L) were calculated using the calibration curves of the standard solution for each pure sugar (Sigma, Aldrich, St. Louis, MO, USA). The CHROMELEON Chromatography Management System was used for chromatogram acquisition. The concentrations of the identified sugars (fructose, glucose, sucrose, turanose, trehalose, and maltose) were expressed as g/100 g of honey.

### 2.8. Statistical Analysis

Multivariate statistical analysis was applied to identify differences and similarities between samples. IBM SPSS statistics software 23.0 (IBM, Armonk, NY, USA) and Statgraphics centurion V18 (The Plains, USA) were used. First, one-way ANOVA was carried out using the bioclimatic area as the factor, and the main pollen grains (those that presented values over 3% of the pollen spectra), physicochemical parameters, bioactive compounds, and sugars as variables. Differences between groups were tested through post hoc comparison using the Bonferroni test. Significance was calculated for *p* < 0.05. Lastly, an exploratory technique like principal-component analysis (PCA) was performed. This technique allows to reduce dimensionality in data, increasing the visibility of the relationship between introduced variables in analysis. The used variables were the same as those mentioned in ANOVA. Samples were considered as cases and marked according to their botanical origin.

## 3. Results and Discussion

### 3.1. Pollen Spectra and Content of Honey Samples

The samples were from a large area of northern Algeria (Table 1). We collected 33 samples in the northwest and west of the country, 13 in the north (central part), 14 in the east and northeast, and 2 from southern areas. Sampling covered different bioclimatic regions, the most common being the semiarid region that provided 34 samples, 15 were from Mediterranean subhumid areas, 4 from humid areas (with more than 1000 mm of annual rain), and 9 from arid areas with annual precipitation below 400 mm. Most samples were produced in Tell (42 samples), the most populated region, where agrosystems and forest areas are common. Fifteen were from the steppes of the high plateau, and 5 from the Sahara.

Palynological analysis of the samples showed high diversity in the plants represented in the pollen spectra. Most samples contained more than 20 different pollen grains, and some samples even more than 30.

A total of 104 pollen types belonging to 51 botanical families were identified in the 62 samples. Pollen types *Eucalyptus*, *Olea europaea*, *Brassica napus*, *Echium*, *Ziziphus lotus*, *Papaver rhoeas*, *Genista*, *Foeniculum*, *Hedysarum*, *Tamarix*, *Eryngium campestre*, and *Pimpinella anisum* were presented in more than 50% of the samples. Some of them reached maximal values in pollen spectra higher than 80%. These were *Eucalyptus*, *O. europaea*, *Z. lotus*, and *Foeniculum*. Other common dominant pollen types (≥45% of pollen spectra) were *B*. *napus* (maximum of 69.0%), *Genista* type (58.8%), *Tamarix* (58.1%), *Eryngium campestre* (52.1%), and *Hedysarum* (45.9%) (Table 2). Sporadically, other pollen types were dominant: *Eriobotrya* (75.4%), *Eruca sativa* (75.1%), *Melilotus* (68.7%), *Punica granatum* (56.2%), and *Erica arborea* (55.1%). The rest of the pollen types were always identified with percentages below 45% of the pollen spectra. Some samples stood out with secondary pollen grains from the Arecaceae family, such as *Chamaerops* (maximum value of 37.3%), *Capparis* (maximum of 36.7%), *Asparagus* (34.3%) or *Paronychia argentea* (maximum of 28.2%).

Quantitative pollen analysis showed 3 samples (pressed honey) with pollen content higher than 100,000 grains of pollen per gram of honey. Two samples had a pollen content between 50,000 and 100,000 pollen grains/g honey (Class IV of Maurizio), 16 were from Class III (10,000–50,000 pollen grain/g honey), 30 had Class II pollen content (2000–10,000 pollen grain/g honey), and 11 a pollen content lower than 2000 pollen grain/g honey (Class I).

### 3.2. Sample Palynological Profile Regarding Bioclimatic Areas of Origin

**Figure 2:**
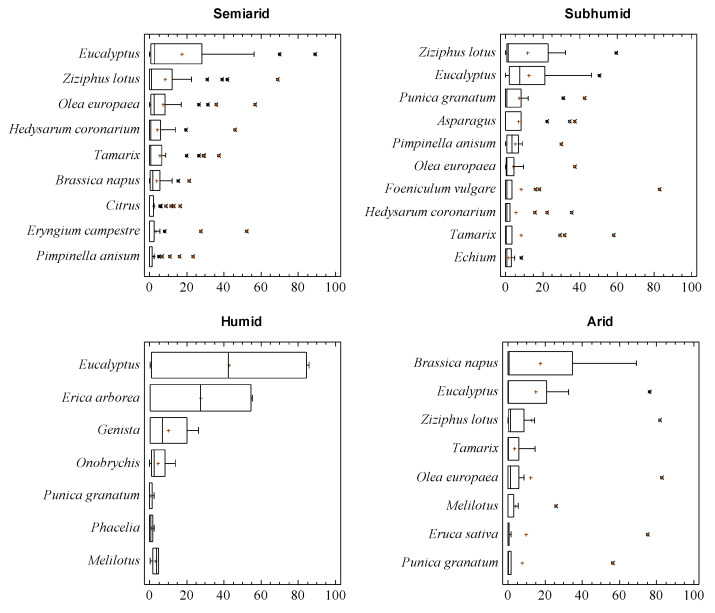
Box and Whisker plot of the main pollen types in each area. * Atypical values, + Centroids.

*Eucalyptus*, *Z. lotus* and some *Apiaceae* such as *Foeniculum* or *Eryngium* pollen types were the dominant pollen in many samples from the semiarid and subhumid Tell regions. Other pollen types, such as *Tamarix* or *Hedysarum* were also common in these samples (Figure 2). Less common pollen types, such as *P. granatum* or *Eriobotrya*, were also found as dominant pollen in samples from this origin. The first appeared in three summer honey samples, and the second one in a single sample collected in winter. There stood out 11.1% of samples with high percentages of *O. europaea* pollen, sometimes over 80% of pollen spectra. This was mainly associated with pressed honey and samples that are extraordinarily rich in pollen grains, indicating that this procedure introduces high quantities of pollen grains in honey. Accompanying or important pollen types were found, pollen grains from herbaceous plants common in agrosystems like *Hedysarum*, *Melilotus*, *Onobrychis*, *B. napus*, *Phacelia*, *Echium* or *Melilotus*. Other well-represented pollen types were those from *Apiaceae* such as *Thapsia* or *Pimpinella*. Plants from semiarid and salted lands, named *Tamarix* or *P. argentea*, were frequent in many samples. One of the most representative pollen grains corresponded to *Citrus*, which determined the botanical origin of some samples. In addition to this, *Asparagus* pollen was found with high percentages in samples from Tizi Ouzou and Tlemcen, and *Acacia* in Mostaganem samples. The apicultural value of some *Acacia* species should be studied even though it is considered non-nectariferous. Nectar secretion was found in different *Acacia* species [27] and some *Acacia* spp. honey from different countries were studied [28,29].

Regarding honey samples from humid areas, two samples stood out from the El Taref region with *Erica arborea* as the dominant pollen. Samples from the high plateau contained as the dominant pollen *Apiaceae*-like *Eryngium campestre* type, *Hedysarum* or *Ziziphus lotus*, and, as accompanying or important pollen, a great diversity of *Apiaceae*, such as *Foeniculum*, *Pimpinella*, *Thapsia*, Asteraceae such as *Carduus*, *Centaurea*, or *Cichorium* type. The presence of *Chamaerops* was considered high. Samples from the M’Sila region had *B. napus* as dominant pollen.

Samples from the arid region had as the dominant pollen *P. granatum* (the sample collected in Laghouat) and *Z. lotus* or *Eruca sativa* (honey from the Naama region). The sample from Bechar was considered to be from *Citrus*.

### 3.3. Quality and Physicochemical Sample Properties

Samples showed important differences in the studied physicochemical parameters. Humidity varied from 14.4% to 22.5%. This maximal value corresponded to a sample collected in El Taref (the humid region). Electrical conductivity was from the 0.133 mS/cm of a sample collected in Sidi bel Abbés to the 1.460 mS/cm of a sample from Naama; pH was from 3.5 (El Taref sample) to 4.7 (Médea sample). Parameters related to sample freshness also had great variation. HMF was higher than 40 mg/kg in two samples, showing the bad quality of these samples. The rest of the samples presented lower values, frequently near 10 mg/kg, with the lowest being 1 mg/kg. Following this trend, diastase activity was low in samples with high HMF content. Excepting these samples, the lowest value was 8.9° and the highest was 40.6°. The high HMF content of some Algerian honey samples (over the limit of 40 mg/kg established in international quality schemes) was noted before [30]. Improvement of the management practices is necessary to avoid this situation. An important parameter for the first differentiation of samples is color, which varied from 13 (Tebessa sample) to 150 mm Pfund (Tizi Ouzou sample) (samples with higher HMF content were not considered for mean values of color, diastase content, and HMF content).

Most of the samples had a sugar content according to blossom honey, this being a sum of fructose and glucose higher than 60%. The lowest value corresponded to a honeydew sample (52.8%), while the highest value was 75.6%. Fructose content varied from 33.0% in a Tebessa sample to 44.4% in a Guelma sample. Glucose oscillated from 23.1% to 34.1%. The concentration of other sugars was really low. Maltose varied from 0.6% to 3.6%, turanose from 1.0% to 3.1%, and raffinose had a highest value of 3.2%. Lastly, sucrose had the highest value (3.3%) in a *Citrus* honey, but was only found in 20 samples.

Other important compounds, related to healthy properties of honey, were polyphenols and flavonoids. The first varied from 20.0 mg/100 g in a sample of *E. japonica* from Sidi bel Abbes to 182.3 mg/100 g of a honeydew sample from M’Sila. Flavonoid content varied from 1.0 mg/100 g to 12.9 mg/100 g in a sample from Bechar and a sample from Tlemcen, respectively. Moreover, RSA was greatly diverse, this variation being from 10% to 79.5%.

### 3.4. Influence of Area of Origin on Sample Characteristics

ANOVA was performed to mark differences and similarities between sample characteristics regarding their bioclimatic region. The highest differences were found between samples from humid areas, situated in the NE of the country, and samples collected in semiarid, subhumid, and arid areas. The most significant (*p* < 0.05) were higher electrical conductivity (mean value of 0.910 mS/cm), darker color (mean value of 122 mm Pfund), high water content (mean value of 21.4%), higher flavonoid content, and lower fructose content (mean value) in samples from humid areas in comparison with the others. In ANOVA results, the higher content in *E. arborea* stood out, together with *Malus* and *Onobrychis* types for honey of this origin. Furthermore, samples from semiarid, subhumid, and arid regions did not show significant differences (*p* < 0.05) between them in physicochemical variables except for lower sample humidity from those of the arid region (mean value of 15.7%). With regard to pollen spectra, samples from arid areas had higher content of *B. napus* and *Rosmarinus* than those from semiarid and subhumid regions (*p* < 0.05). However, due to the great variation between samples, more studies are needed.

### 3.5. Botanical Characterization of Honey Samples

The botanical origin of samples is the most important attribution to valorize local honey production. Sample typification was done with information provided by beekeepers, physicochemical data, and sample pollen profiles. To calculate the percentage of the main pollen type in the samples, nectarless plants were excluded. The sample classification and physicochemical data of each honey type are shown in Table 3.

The main group was polyfloral honey with 26 samples. This type of honey is produced in all regions, presenting significant variations in the studied variables. The pollen spectra had as main pollen types *Eucalyptus* and *Ziziphus*, but *Apiaceae* (mainly *Foeniculum*, *Eryngium*, *Pimpinella*, and *Thapsia* pollen types), *Hedysarum*, *Melilotus*, *Centaurea*, *B. napus*, or *Echium*) were common. Pollen grains from plants growing in semiarid and arid areas, such as *Tamarix* or *Capparis spinosa*, were also present in relevant amounts. Five samples produced in Medea, Mostaganem, and Tlemcen had a pollen content higher than 30% of *Tamarix* pollen, but their physicochemical properties, and polyphenol and flavonoid content strongly varied between samples. The apicultural value of *Tamarix* is not well-known; while some authors considered this plant important for honey production [31], other authors indicated that the nectar concentration in flowers of *Tamarix* plants is marginal [32]. Regarding *Capparis spinosa*, their pollen grains reached percentages over 20% in one sample from Sidi bel Abbes and another from Medea (36.7% and 24.4%, respectively). Important nectar production for this plant was indicated in a few studies [33,34], but this honey type is little mentioned in the scientific literature [13,29,35]. Another interesting point regards two samples with a high quantity of *Asparagus* pollen type (22.1% and 34.3%); both were from the Tizi Ouzou region and presented similar patterns in the studied variables. The most significant was the low pollen content (less than 2000 pollen grains/g of honey), the color (value close to 90 mm Pfund), diastase content (more than 30° Gothe), and polyphenol (mean value of 100 mg/100 g) and flavonoid content (mean value of 7 mg/100 g). There is little information about the importance of Asparagaceae plants for honey production, but the pollination activity and pollen production in both female and male plants of *Asparagus* was constated [36]. All samples mentioned before were considered polyfloral, but further studies are needed to endorse possible monovarietal honey.

Regarding the number of pollen types, polyfloral samples had a high number with a mean value of 25, but frequently with more than 30 pollen types. The values on physicochemical parameters, sugar profile, and antioxidant components were like those of other polyfloral honey types studied in the country [8,13].

Seven samples were typified as *Eucalyptus* honey, with a mean value of this pollen of 76.7%. Two samples were pressed honey having a high amount of pollen. The rest had a mean value included in Class III of Maurizio. This is slightly high pollen content in comparison with that of *E. globulus* honey from the Atlantic area of Europe [37]. Some of these samples presented high humidity with a mean value of 19.4%. In comparison with *Eucalyptus* honey from other areas, the studied samples presented high electrical conductivity, color, and pH [3,37,38]. This sample type is mainly produced in forests of coastal areas during summer, so some honeydew supports can influence their physicochemical attributes. *Eucalyptus* samples are produced in the Mostaganem region and eastern areas of the country. In Algeria, *Eucalyptus* trees were introduced in 1850, with *E. camaldulensis* and *E. globulus* being the main introduced species. In the middle of S. XX, large areas of the southeast, center, and west, such as Mostaganem, were intensively planted with *Eucalyptus* for wood production. Currently, species such as *E. gomphocephala*, *E. sideroxylon*, *E. robusta*, or *E. viminalis* grow there [39].

Six samples from the regions of Tizi Ouzou, Tlemcen, M’Sila, and Naama were typified as honeydew honey, while beekeepers had named them forest honey. Samples had the highest electrical conductivity, polyphenol content, and dark color with the lowest sum of fructose and glucose, common features to other types of honeydew honey [40,41,42,43]. Little information is available about the honeydew production in Algeria, and knowledge about sources of this honey is scarce. However, it was the subject of a great number of recent studies in other territories [40,41,42,43,44,45,46]. Considering the interest of this honey type, and that differentiation between different types of honeydew honey or even other dark blossom honey is difficult [47], more studies regarding this honey type could be useful to valorize local honey production.

Another important source for honey production was *Apiaceae* plants. A total of five samples were typified within this group. Three samples had as the main pollen the *F. vulgare* type, one sample *P. anisum*, and another *E. campestre* type. Samples were included in a single group due to some of them sharing high percentages of the mentioned pollen types and other secondary pollen types, such as *Thapsia* or *Apium* showing the importance for the honey production of this botanical family in the area. In general, samples were characterized by electrical conductivity near 0.495 mS/cm, light amber color, and high fructose content; the sample from *P. anisum* had a darker color. These samples had high polyphenol content (among the highest) and with medium flavonoid content. *Apiaceae* honey from North African countries was mentioned in different papers, mainly from plants as *Eryngium campestre*, *Ammi visnaga*, *Ridolfia segetum*, or *Bupleurum spinosum* in Morocco [48,49]. Some *Apiaceae* from Algeria were studied regarding sugar composition or polyphenol content [12,21]. The studied fennel honey had electrical conductivity, diastase content, and sugar content similar to those from Tenerife samples [50]. A high amount of hydroxycinnamic acids, mainly caffeic acid, was mentioned for this honey type [51].

*Citrus* samples are produced mainly in the Tell region (Mostaganem, Mascara, and Relizane), where these fruit plants are common in agricultural lands, but also in oases of arid areas (Bechar). Five samples were considered from this botanical origin. The pollen of *Citrus* was identified with a mean value of 22.0% (nectarless pollen excluded), less than 15% of pollen spectra. It is common to find this pollen type with other pollen types such as *O. europaea*, *Genista*, or *Tamarix*. Samples had extralight amber color, mean electrical conductivity of 0.318 mS/cm, and high fructose and glucose content besides the highest mean sucrose content. These samples had similarities with other studied samples from Blida region, such as the presence of high percentages of *O. europaea*, and similar values in electrical conductivity, fructose, and glucose content [8,52].

Four samples were considered unifloral sedra honey. The most important species in the area to produce this type of honey is *Z. lotus*. The samples had light amber color, mean electrical conductivity of 0.535 mS/cm, high pH, and medium polyphenol and flavonoid contents. Sedra honey is more known than other monofloral honey types, as consumers appreciate them much due to healthy properties attributed to them [53]. The characteristics of the honey type are in accordance with the results of other publications [19,29].

The typification of three samples as pomegranate samples (*P. granatum*) stands out. The production of this honey type is not mentioned in the country, as it is rare. *P. granatum* pollen was presented in a mean value of 53.3%, and samples had medium-to-low pollen content (Class II of Maurizio). Water content was low (mean of 14.9%), color was extralight amber, the mean of electrical conductivity was of 0.580 mS/cm, and polyphenol and flavonoid contents were medium.

Two samples from El Taref were typified as heather honey, with *E. arborea* having a mean value of 55.7%. Northwestern evergreen forests and high shrubs growing in humid and warm climates facilitate the production of this honey type. Samples presented high humidity content (mean value 20%), high electrical conductivity, and the darkest color. Furthermore, polyphenol content was high, and samples presented major flavonoid content (mean of 11.1 mg/100 g). However, *E. arborea* honey remains produced in the area is poorly described [12].

Lastly, one sample was typified as unifloral from *Retama sphaerocarpa* (*Genista* pollen type was present in 79.9% of the pollen spectra), one sample as *Rosmarinus* (*Rosmarinus* pollen percentage was 17.0%), one sample as medlar honey (*E. japonica* pollen content was 75.4%), and one as sulla honey, with a pollen content of *Hedysarum* of 49.3%. Samples from *Rosmarinus*, *Eriobotrya*, and *Hedysarum* had the clearest color, the first being an extrawhite honey, and the last two white honey. The best-described honey type is *Hedysarum* honey [2,7,12].

**Figure 3:**
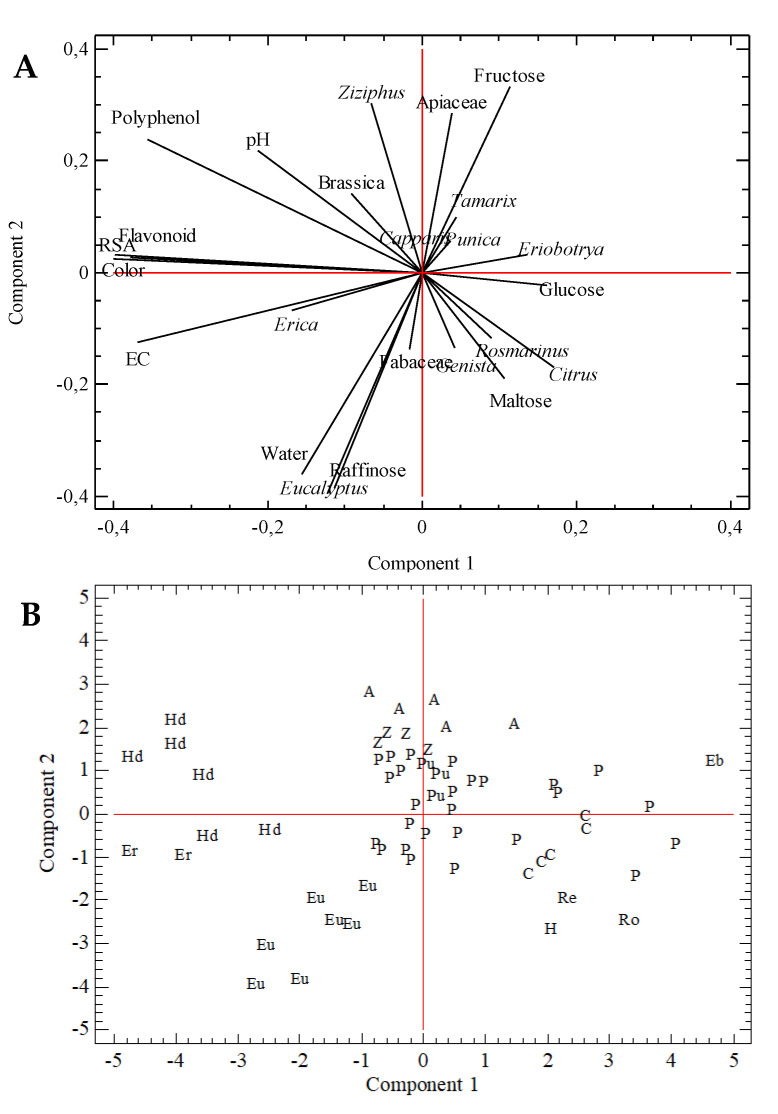
Principal-component analysis. (**A**) Loading plot of variables; (**B**) Score plot of the first two extracted components and sample botanical origin. A, *Apiaceae*; C, *Citrus*; Eb, *Eriobotrya*; Er, *Erica*; Eu, *Eucalyptus*; Hd, honeydew; H, *Hedysarum*; P, polyfloral; Pu, *Punica*; Re, *Retama*; Ro, *Rosmarinus*; Z, *Ziziphus*.

As commented before, samples presented a wide variation in physicochemical parameters and pollen content. To reduce the dimensionality of the dataset and increase the interpretability of results, PCA was applied. The first nine components explained 72% of data variance, corresponding the sum of Components 1 and 2 to 30%. The plot of the scores of these two components is included in Figure 3A. Left is flavonoid content, RSA, and color close to electrical conductivity (negative quadrant) and polyphenol content (positive quadrant). *Erica* pollen is situated in the negative quadrant in the left with the electrical conductivity. Right shows *Eriobotrya* near glucose. Some pollen variables, such as *Punica*, *Capparis*, or *Tamarix*, were in the same direction as that of Fructose and *Apiaceae*. *Ziziphus*, *Brassica*, and pH are situated in the same quadrant as that of polyphenol content. On the opposite quadrant are *Citrus* and *Rosmarinus* pollen types, close to Maltose. Lastly, at the bottom, *Fabaceae* close to *Eucalyptus*, humidity, and raffinose sugar. PCA grouped samples from the same botanical origin (Figure 3B). Left are honeydew samples (Hd), *Erica* (Er), and *Eucalyptus* (Eu). This position corresponds to samples with the highest humidity content (*Eucalyptus* and *Erica* samples), major electrical conductivity, and darker color. The influence of polyphenol content determined the situation of *B. napus* in this area, as samples with a high content of this pollen type were considered honeydew honey due to their color, electrical conductivity, and the polyphenol content. In the top of the figure are *Apiaceae* (A) samples and *Z. lotus* (Z) honey, and in the center are *P. granatum* samples (Pu), close to some polyfloral samples. This sample group was separated from other, more disperse groups, situated in the left, which included types *Citrus* (C), *Hedysarum* (H), *Rosmarinus* (Ro), and *R. sphaerocarpa* honey. The *Eriobotrya* sample was clearly separated.

PCA results showed sample similarities classified with the same botanical origin, but also variation in some of these sample groups. This was the case of samples from *Eucalyptus*, honeydew, and, to a lesser extent, *Apiaceae* or *Citrus* samples. On the other hand, common Mediterranean honey types (*Citrus*, *Rosmarinus*, *Hedysarum*, or *Retama*) were close, evidencing the similarities between them.

## 4. Conclusions

The results of this paper showed the great variability in local honey production of Algerian beekeeping, and the potentiality regarding different honey types that could be obtained. Some of the plant species mentioned in this work, such as *Eucalyptus*, *Brassica napus*, *Hedysarum*, and *Citrus*, are common honey plants in Mediterranean areas. However, others, such as *Capparis spinosa*, *Asparagus*, *Tamarix*, *Ziziphus lotus*, and some *Apiaceae* plants (*Eryngium*, *Thapsia*, *Pimpinella*), even *Acacia*, are representative of the honey of this country, useful as markers to guarantee their authenticity. In any case, there is little information on the apicultural value of some of them and of their honey; thus, more research is needed on these topics.

## Figures and Tables

**Table 1:** Geographical origin of samples, climate, and harvest period.

Localities	Geographical Situation	Climate	Harvest Period	No. Samples
Bechar	SW, Sahara	Arid	Summer	1
Laghouat	S, Sahara	Arid	Summer	1
M’Sila	E, High plateau	Arid	Summer	2
Naama	W, Sahara	Arid	Summer	3
Tebessá	E, High plateau	Arid	Spring, Summer	2
El Taref	NE, Tell	Humid	Summer	4
Boumerdes	N, Tell	Semiarid	Summer	1
Mascara	NW, Tell	Semiarid	Summer	2
Mostaganem	NW, Tell	Semiarid	Spring, Summer	15
Relizane	NW, Tell	Semiarid	Summer	2
Saida	NW, High plateau	Semiarid	Summer	3
Sidi Bel Abbés	NW, Tell	Semiarid	Summer, Winter	3
Souk-Ahras	NE, High plateau	Semiarid	Summer	1
Médea	N, High plateau	Semiarid	Summer	7
Blida	N, Tell	Subhumid	Spring	2
Guelmá	NE, Tell	Subhumid	Summer	5
Tizi-Ouzou	N, Tell	Subhumid	Summer	3
Tlemcen	NW, Tell	Subhumid	Summer	5

**Table 2:** Main palynological characteristics regarding geographical origin.

Localities	Dominant Pollen (≥45%)	Accompanying Pollen (45–15%)	Important Pollen (15–3%)	Other Pollen (3–1%)
Bechar (*n* = 1)	*Olea* (82.8% ^1^)/1 ^2^		*Tamarix, Citrus*	*Sinapis*
Blida (*n* = 2)	*Foeniculum* (82.8%)/1 *Eucalyptus* (46.1%)/1	*Hedysarum* (35.4%)	*Pimpinella, Echium, Genista, Punica, Citrus*	*Paronychia, Brassica*
Boumerdes (*n* = 1)	-	*Pimpinella* (23.3%), *Ziziphus* (17.5%)	*Thapsia, Apium, Eryngium, Echium, Hedysarum, Eucalyptus, Tamarix*	*Brassica*
El Taref (*n* = 4)	*Eucalyptus* (85.7%)/2 *Erica* (55.1%)/1	*Genista* (26.3%)	*Melilotus, Onobrychis, Malus*	*Foeniculum, Trifolium. Papaver, Punica, Phacelia*
Guelmá (*n* = 5)	*Eucalyptus* (50.5%)/1	*Olea* (37.2%), *Ziziphus* (22.7%), *Punica* (42.4%), *Hedysarum* (15.5%), *Pimpinella* (29.8%), *Phacelia* (17.6%), *Myrtus* (17.3%)	*Eryngium*, *Thapsia*, *Carduus*, *Borago*, *Echium*, *Erica, Genista*, *Melilotus*, *Ononis*, *Trifolium*, *Papaver*, *Malus*, *Ailanthus*	*Pistacia*, *Artitalicisia*, *Brassica*, *Onobrychis*, *Peganum*, *Capparis*
Laghouat (*n* = 1)	*Punica* (56.2%)/1		*Ziziphus, Olea*	*Acacia*, *Centaurea*, *Brassica*
M’Sila (*n* = 2)	*Brassica* (69.03%)/2	*Galega* (21.1%)	*Artitalicisia*, *Rosmarinus*, *Thymus*, *Capparis*	*Euphorbia*, *Ziziphus*, *Daphne*
Mascara (*n* = 2)	*Eucalyptus* (69.9%)/1 Olea (56.8%)/1	*Tamarix* (26.3%)	*Genista*, *Hedysarum*, *Quercus*, *Citrus*	*Pistacia*, *Thapsia*, *Melilotus*, *Chamaerops*, *Prunus*
Médea (*n* = 7)	*Eryngium* (52.1%)/1	*Ziziphus* (41.5%), *Melilotus* (15.6%), *Tamarix* (37.0%), *Eryngium* (27.6%), *Hedysarum* (19.4%), *Pimpinella* (16.1%), *Eucalyptus* (43.0%)	*Foeniculum*, *Pimpinella*, *Thapsia*, *Artitalicisia*, *Carduus*, *Centaurea*, *Cichorium*, *Echium*, *Brassica*, *Chenopodium*, *Convolvulus*, *Genista*, *Onobrychis*, *Quercus*, *Olea*, *Papaver*, *Capparis*	*Apium*, *Thymus*, *Cistus*, *Cyperus*, *Euphorbia*, *Allium*, *Chamaerops*, *Phacelia*
Mostaganitalic (*n* = 15)	*Eucalyptus* (88.9%)/4 *Genista* (58.8%)/1 *Punica* (48.6%)/1	*Olea* (35.7%), *Tamarix* (19.8%), *Melilotus* (24.8%), *Genista* (30.8%), *Capparis* (24.4%), *Paronychia* (28.2%), *Brassica* (15.2%)	*Schinus*, *Foeniculum*, *Centaurea*, *Chrysantitalicum*, *Echium*, *Sinapis*, *Buxus*, *Convolvulus*, *Acacia*, *Ceratonia*, *Hedysarum*, *Muscari*, *Chamaerops*, *Papaver*, *Ziziphus*, *Rubus*, *Citrus*, *Ailanthus*	*Melia, Smilax*
Naama (*n* = 3)	*Ziziphus* (81.5%)/1 *Eruca* (75.1%)/1	*Eucalyptus* (32.5%), *Melilotus* (25.6%)	*Pimpinella, Olea, Tamarix*	*Apium*, *Eryngium*, *Acacia*, *Genista*, *Chamaerops*, *Punica*, *Peganum*, *Capparis*
Relizane (*n* = 2)	*Genista* (45.6%)/1	*Foeniculum* (38.3%), *Citrus* (16.3%), *Ziziphus* (38.8%)	*Ammi, Eryngium, Sinapis, Olea, Tamarix*	*Convolvulus, Galega*
Saida (*n* = 3)	*Ziziphus* (68.9%)/1	*Eucalyptus* (19.7%), *Chamaerops* (17.6%), *Capparis* (36.7%), *Echium* (18.2%), *Centaurea* (22.0%)	*Tamarix*, *Papaver*, *Olea*, *Asparagus*, *Hedysarum*, *Cistus*, *Echium*, *Cichorium*	*Genista*, *Brassica*
Sidi Bel Abbés (*n* = 3)	*Eriobotrya* (75.4%)/1 *Melilotus* (68.7%)/1	*Globularia* (16.5%), *Eucalyptus* (17.8%), *Tamarix* (28.9%)	*Brassica, Ceratonia, Ziziphus*	*Artitalicisia*, *Chrysanthitalicum*, *Sinapis*, *Euphorbia*, *Rosmarinus*, *Olea*
Souk-Ahras (*n* = 1)	*Eucalyptus* (76.1%)/1		*Melilotus, Ononis*	*Ammi, Erica, Punica*
Tebessá (*n* = 2)	*Hedysarum* (45.9%)/1	*Brassica* (21.3%)	*Eryngium, Foeniculum, Sinapis, Lotus, Quercus, Eucalyptus*	*Apium*, *Casuarina*, *Ephedra*, *Olea*, *Malus*, *Citrus*, *Capparis*
Tizi-Ouzou (*n* = 4)	-	*Asparagus* (34.3%), *Hedysarum* (22.2%), *Ziziphus* (32.1%), *Foeniculum* (17.9%)	*Pimpinella*, *Carduus*, *Echium*, *Thymus*, Other *Lamiaceae*, *Olea*, *Papaver*, *Rubus*, *Castanea*	*Apium*, *Eryngium*, *Thapsia*, *Chrysantitalicum*, *Cichorium*, *Brassica*, *Cistus*, *Ononis*, *Quercus*
Tlitaliccen (*n* = 5)	*Ziziphus* (59.5%)/1 *Tamarix* (58.1%)/1	*Eucalyptus* (23.2%), *Punica* (30.9%), *Chamaerops* (37.3%)	*Schinus*, *Eryngium*, *Foeniculum*, *Pimpinella*, *Chenopodium*, *Ceratonia*, *Olea*, *Peganum harmala*, *Capparis*	*Pistacia*, *Artitalicisia*, *Taraxacum*, *Arctium*, *Brassica*, *Cistus*, *Hedysarum*, *Muscari*, *Papaver*
Bechar (n = 1)	*Olea* (82.8% ^1^)/1 ^2^		*Tamarix, Citrus*	*Sinapis*
Blida (n = 2)	*Foeniculum* (82.8%)/1 *Eucalyptus* (46.1%)/1	*Hedysarum* (35.4%)	*Pimpinella, Echium, Genista, Punica, Citrus*	Paronychia, Brassica
Boumerdes (n = 1)	-	Pimpinella (23.3%), Ziziphus (17.5%)	Thapsia, Apium, Eryngium, Echium, Hedysarum, Eucalyptus, Tamarix	Brassica
El Taref (n = 4)	Eucalyptus (85.7%)/2 Erica (55.1%)/1	Genista (26.3%)	Melilotus, Onobrychis, Malus	Foeniculum, Trifolium. Papaver, Punica, Phacelia
Guelmá (n = 5)	Eucalyptus (50.5%)/1	Olea (37.2%), Ziziphus (22.7%), Punica (42.4%), Hedysarum (15.5%), Pimpinella (29.8%), Phacelia (17.6%), Myrtus (17.3%)	Eryngium, Thapsia, Carduus, Borago, Echium, Erica, Genista, Melilotus, Ononis, Trifolium, Papaver, Malus, Ailanthus	Pistacia, Artemisia, Brassica, Onobrychis, Peganum, Capparis
Laghouat (n = 1)	Punica (56.2%)/1		Ziziphus, Olea	Acacia, Centaurea, Brassica
M’Sila (n = 2)	Brassica (69.03%)/2	Galega (21.1%)	Artemisia, Rosmarinus, Thymus, Capparis	Euphorbia, Ziziphus, Daphne
Mascara (n = 2)	Eucalyptus (69.9%)/1 Olea (56.8%)/1	Tamarix (26.3%)	Genista, Hedysarum, Quercus, Citrus	Pistacia, Thapsia, Melilotus, Chamaerops, Prunus
Médea (n = 7)	Eryngium (52.1%)/1	Ziziphus (41.5%), Melilotus (15.6%), Tamarix (37.0%), Eryngium (27.6%), Hedysarum (19.4%), Pimpinella (16.1%), Eucalyptus (43.0%)	Foeniculum, Pimpinella, Thapsia, Artemisia, Carduus, Centaurea, Cichorium, Echium, Brassica, Chenopodium, Convolvulus, Genista, Onobrychis, Quercus, Olea, Papaver, Capparis	Apium, Thymus, Cistus, Cyperus, Euphorbia, Allium, Chamaerops, Phacelia
Mostaganem (n = 15)	Eucalyptus (88.9%)/4 Genista (58.8%)/1 Punica (48.6%)/1	Olea (35.7%), Tamarix (19.8%), Melilotus (24.8%), Genista (30.8%), Capparis (24.4%), Paronychia (28.2%), Brassica (15.2%)	Schinus, Foeniculum, Centaurea, Chrysantemum, Echium, Sinapis, Buxus, Convolvulus, Acacia, Ceratonia, Hedysarum, Muscari, Chamaerops, Papaver, Ziziphus, Rubus, Citrus, Ailanthus	Melia, Smilax
Naama (n = 3)	Ziziphus (81.5%)/1 Eruca (75.1%)/1	Eucalyptus (32.5%), Melilotus (25.6%)	Pimpinella, Olea, Tamarix	Apium, Eryngium, Acacia, Genista, Chamaerops, Punica, Peganum, Capparis
Relizane (n = 2)	Genista (45.6%)/1	Foeniculum (38.3%), Citrus (16.3%), Ziziphus (38.8%)	Ammi, Eryngium, Sinapis, Olea, Tamarix	Convolvulus, Galega
Saida (n = 3)	Ziziphus (68.9%)/1	Eucalyptus (19.7%), Chamaerops (17.6%), Capparis (36.7%), Echium (18.2%), Centaurea (22.0%)	Tamarix, Papaver, Olea, Asparagus, Hedysarum, Cistus, Echium, Cichorium	Genista, Brassica
Sidi Bel Abbés (n = 3)	Eriobotrya (75.4%)/1 Melilotus (68.7%)/1	Globularia (16.5%), Eucalyptus (17.8%), Tamarix (28.9%)	Brassica, Ceratonia, Ziziphus	Artemisia, Chrysanthemum, Sinapis, Euphorbia, Rosmarinus, Olea
Souk-Ahras (n = 1)	Eucalyptus (76.1%)/1		Melilotus, Ononis	Ammi, Erica, Punica
Tebessá (n = 2)	Hedysarum (45.9%)/1	Brassica (21.3%)	Eryngium, Foeniculum, Sinapis, Lotus, Quercus, Eucalyptus	Apium, Casuarina, Ephedra, Olea, Malus, Citrus, Capparis
Tizi-Ouzou (n = 4)	-	Asparagus (34.3%), Hedysarum (22.2%), Ziziphus (32.1%), Foeniculum (17.9%)	Pimpinella, Carduus, Echium, Thymus, Other Lamiaceae, Olea, Papaver, Rubus, Castanea	Apium, Eryngium, Thapsia, Chrysantemum, Cichorium, Brassica, Cistus, Ononis, Quercus
Tlemcen (n = 5)	Ziziphus (59.5%)/1 Tamarix (58.1%)/1	Eucalyptus (23.2%), Punica (30.9%), Chamaerops (37.3%)	Schinus, Eryngium, Foeniculum, Pimpinella, Chenopodium, Ceratonia, Olea, Peganum harmala, Capparis	Pistacia, Artemisia, Taraxacum, Arctium, Brassica, Cistus, Hedysarum, Muscari, Papaver

^1^ The percentage is the maximum value reached for the pollen type, ^2^ Number of samples in which the pollen type is dominant pollen.

**Table 3:** Characteristics of the different honey types. Values are expressed as mean and standard deviation.

	Polyfloral (*n* = 26)	*Eucalyptus* (*n* = 7)	Honeydew (*n* = 6)	*Apiaceae * (*n* = 5)	*Citrus* (*n* = 5)	Sedra (*n* = 4)	*Punica* (*n* = 3)	Heather (*n* = 2)	*Retama* (*n* = 1)	*Rosmarinus* (*n* = 1)	Medlar (*n* = 1)	Sulla (*n* = 1)
Main pollen (%)		*Eucalyptus * (76.7 ± 9.3)		*Apiaceae* (59.0 ± 19.5)	*Citrus * (22.0 ± 6.3)	*Z. lotus * (52.5%)	*P. granatum * (53.3%)	*E. arborea * (55.7 ± 0.6)	*Genista * 79.9%	*Rosmarinus * 17.0%	*Eriobotrya * 75.4%	*Hedysarum * 49.3%
N. Pollen types	25 ± 6	19 ± 6	23 ± 8	23 ± 6	18 ± 6	26 ± 7	22 ± 3	20 ± 2	12	23	13	23
PK (pollen/g)	8526 ± 13,738	27,985 ± 22,930	7433 ± 5757	5155 ± 3674	9935 ± 5787	16,775 ± 10,200	3612 ± 2033	23,750 ± 17,182	21,925	1575	6125	6750
Humidity (%)	16.6 ± 1.3	19.4 ± 2.0	17.2 ± 0.7	16.2 ± 1.0	17.3 ± 1.6	16.6 ± 1.0	14.9 ± 0.2	20.0 ± 0.2	18.9	16	14.6	15.2
EC (mS/cm)	0.481 ± 0.2	0.825 ± 0.2	1.033 ± 0.231	0.495 ± 0.2	0.318 ± 0.1	0.535 ± 0.1	0.580 ± 0.1	0.956 ± 0.2	0.440	0.330	0.135	0.133
pH	3.9 ± 0.2	4.0 ± 0.2	4.2 ± 0.2	4.0 ± 0.3	3.9 ± 0.2	4.4 ± 0.3	4.1 ± 0.1	4.1 ± 0.1	3.6	3.8	3.9	4.0
Color (mm Pfund)	73 ± 22	96 ± 10	125 ± 13	78 ± 19	45 ± 7	79 ± 9	77 ± 7	141 ± 3	67	13	28	34
DI (° Ghote)	20.1 ± 8.9	18.0 ± 6.6	21.5 ± 8.1	25.7 ± 5.0	18.7 ± 6.3	23 ± 3.2	14.1 ± 6.7	14.0 ± 6.6	28.3	6.4	20.6	6.7
HMF (mg/100 g)	1.7 ± 0.9	1.2 ± 0.4	1.7 ± 1.5	1.4 ± 1.1	1.3 ± 0.6	0.9 ± 0.6	1.4 ± 0.4	1.4 ± 1.9	1.4	0.9	0.7	1.0
Fructose (%)	39.9 ± 2.3	37.8 ± 1.9	38.6 ± 0.8	41.5 ± 0.6	40.8 ± 1.7	39.5 ± 0.7	38.5 ± 0.4	37.1 ± 0.2	38.2	38.1	40.3	33
Glucose (%)	29.3 ± 2.7	29.8 ± 1.8	28.0 ± 2.6	29.3 ± 2.3	29.3 ± 2.0	28.8 ± 1.8	28.9 ± 0.4	31.2 ± 3.3	31.6	30.1	34.1	25.9
Sacarose (%)	1.1	nd	nd	nd	2.3	nd	0.1	nd	0.7	nd	0.6	1.3
Maltose (%)	2.1 ± 0.6	2.0 ± 0.4	2.2 ± 0.6	1.3 ± 0.3	2.3 ± 0.7	2.1 ± 0.5	2.4 ± 0.4	1.5 ± 0.1	2.4	3.3	2.1	1.8
Turanose (%)	1.7 ± 0.4	2.0 ± 0.4	1.7 ± 0.5	1.8 ± 0.5	1.7 ± 0.3	2.5 ± 0.5	2.0 ± 0.2	1.3 ± 0.1	1.3	3.1	1.7	1.2
Raffinose (%)	0.4 ± 0.6	1.5 ± 1.3	0.4 ± 0.5	0.2 ± 0.1	0.2 ± 0.2	0.2 ± 0.1	0.3 ± 0.1	0.1 ± 0.0	0.1	0.3	0.1	1.5
Polyphenol (mg/100 g)	67.7 ± 22.2	72.7 ± 16.8	141.2 ± 34.1	104.5 ± 10.3	50.5 ± 31.5	71.6 ± 21.3	95.1 ± 5.4	130.8 ± 10.8	48.1	26.5	20	60.1
Flavonoid (mg/100 g)	4.9 ± 1.8	7.1 ± 1.2	10.6 ± 1.4	5.5 ± 0.8	4.1 ± 2.7	5.6 ± 1.0	5.1 ± 1.0	11.1 ± 0.9	1.4	1.0	1.4	5.9
RSA (%)	30.6 ± 12.2	42.4 ± 10.6	61.5 ± 14.7	28.0 ± 9.28	16.8 ± 8.7	31.1 ± 8.8	33.6 ± 8.3	43.7 ± 5.0	22.4	13.3	14.3	17.0

nd: not detected.
